# Editorial: Sense of agency: examining awareness of the acting self

**DOI:** 10.3389/fnhum.2015.00310

**Published:** 2015-06-03

**Authors:** Nicole David, Sukhvinder Obhi, James W. Moore

**Affiliations:** ^1^Department of Neurophysiology and Pathophysiology, University Medical Center Hamburg-EppendorfHamburg, Germany; ^2^Department of Psychology, Neuroscience & Behaviour, McMaster UniversityHamilton, ON, Canada; ^3^Department of Psychology, Goldsmiths, University of LondonLondon, UK

**Keywords:** agency (psychology), volition, action, disorders, consciousness

For a long time interest in the sense of agency was confined to a small group of researchers such as philosophers of mind concerned with fundamental questions of consciousness and free will or neuropsychologists investigating mental illnesses that clearly involve abnormalities of agency (e.g., psychosis). This niche existence obscured the concept's relevance for many societal and cultural phenomena in which individuals experience subjective control over objectively uncontrollable events, or neglect control over events they have caused. It also resulted in a rather small number of scientific studies addressing the sense of agency. Today, scientific investigations of sense of agency constitute a rapidly expanding field. This is evident in the rising number of scientific articles related to the topic as listed on search engine databases such as PubMed.

Significant progress has been made with respect to some fundamental questions concerning the sense of agency, for example, in shedding light on the brain regions supporting sense of agency or in clarifying its conceptual boundaries. Yet, numerous questions remain unanswered. These include, but are not limited to, what neural network dynamics underlie the sense of agency, how does the sense of agency develop across the lifespan (e.g., in children compared to adults), and how can agency research be used in more applied domains, like engineering and computer science. The contributions in this research topic go some way to answering these questions. Here we provide a brief overview of these contributions, focusing on general themes that have emerged.

A number of contributions add to the theoretical literature on sense of agency. Some consider the applicability of Bayesian approaches to sense of agency (Friston et al., 2013; Moutoussis et al., 2014), an exciting development that promises to integrate agency research within a wider theoretical framework for understanding neurocognitive function. Other theoretical contributions have highlighted, and attempted to overcome, problems with existing models of agency processing (Carruthers, 2014; Chambon et al., 2014; Cioffi et al., 2014; Gentsch and Synofzik, 2014; Sowden and Shah, 2014; Swiney and Sousa, 2014). These cover a range of issues such as the affective dimension of agency processing (Gentsch and Synofzik, 2014) and the contribution of prospective (pre-motor) cues to sense of agency (Chambon et al., 2014). Collectively, these contributions demonstrate the relative maturity of theoretical work on sense of agency and how significant progress is being made in our understanding of it. Finally, other theoretical contributions have looked at more applied aspects of agency research, for example, the relevance of agency theory and methods for the field of human-computer-interaction, an exciting new arena in which to explore sense of agency (Limerick et al., 2014).

Amongst the original research articles, a large group of contributions used the so-called Libet-clock to investigate sense of agency, with the majority focusing on the intentional binding effect (Barlas and Obhi, 2013; Cavazzana et al., 2014; Jo et al., 2014; Penton et al., 2014; Pfister et al., 2014; Hascalovitz and Obhi, 2015). Those contributions using intentional binding have done so in new and exciting ways, for example to assess agency processing in children (Cavazzana et al., 2014) and in social contexts (Pfister et al., 2014). However, intentional binding was not the only method used in our empirical contributions and important insights have been gleaned from a number of different methods such as the joint Simon effect and classical psychophysical measures together with Bayesian modeling (Kawabe, 2013; Stenzel et al., 2014). A particularly novel contribution used sensory attenuation to examine agency processing during lucid dreaming, pushing agency research into exciting new areas (Windt et al., 2014).

Additionally, a few contributions further examined the neural underpinnings of the sense of agency in methodologically novel and exciting ways. These have investigated the neural correlates of sense of agency as well as the neural networks supporting this experience (Dogge et al., 2014; Jo et al., 2014; Ritterband-Rosenbaum et al., 2014a,b). These contributions represent a significant advance in neuroimaging approaches to agency processing.

The final theme we have identified in the contributions centers around disorders of agency. These have extended the classical example of psychosis by discussing loss of agency in apraxia, anosognosia for hemiplegia and phantom-limb phenomena (Imaizumi et al., 2014; Pazzaglia and Galli, 2014; Preston and Newport, 2014). There is also an important discussion of the utility of objective measures of sense of agency, such as intentional binding, in helping to improve our understanding of neurological disorders (Wolpe and Rowe, 2014). From these contributions it is becoming increasingly clear that aberrant experiences of agency are an important feature of numerous psychiatric and neurological disorders.

Taken together, this Research Topic demonstrates the impressive breadth of research currently being undertaken on sense of agency. The contributions themselves reveal the various applications, cross-disciplinary relevance and widespread significance of this topic. Sense of agency is now firmly on the agenda of psychologists, philosophers, computer scientists, neuroscientists, and neurologists/psychiatrists. However, despite the fact that significant progress has been made in our understanding of sense of agency and its real-world relevance, there is much work still to be done. Indeed this research topic serves as a record not only of where agency research is at present, but also as an indicator of where it can go in the future.

## Conflict of interest statement

The authors declare that the research was conducted in the absence of any commercial or financial relationships that could be construed as a potential conflict of interest.
